# Magnetic resonance enterographic predictors of one-year outcome in ileal and ileocolonic Crohn’s disease treated with anti-tumor necrosis factor antibodies

**DOI:** 10.1038/srep10223

**Published:** 2015-05-20

**Authors:** Piotr Eder, Michal Michalak, Katarzyna Katulska, Liliana Lykowska-Szuber, Iwona Krela-Kazmierczak, Kamila Stawczyk-Eder, Katarzyna Klimczak, Aleksandra Szymczak, Krzysztof Linke

**Affiliations:** 1 Department of Gastroenterology, Human Nutrition and Internal Diseases, Poznan University of Medical Sciences, Heliodor Swiecicki Hospital, Przybyszewskiego Street 49, 60-355 Poznan, Poland; 2 Department of Computer Science and Statistics, Poznan University of Medical Sciences, Dabrowskiego Street 79, 60-529 Poznan, Poland; 3 Department of General Radiology, Poznan University of Medical Sciences, Heliodor Swiecicki Hospital, Przybyszewskiego Street 49, 60-355 Poznan, Poland

## Abstract

The aim of the study was to assess the role of magnetic resonance enterography (MRE) in predicting one-year efficacy of anti-tumor necrosis factor antibodies - infliximab (IFX), adalimumab (ADA) in Crohn’s disease (CD) patients primarily responding to therapy. We performed retrospective analysis among 61 CD patients who had undergone a successful IFX/ADA induction therapy and were treated with maintenance doses. All patients underwent MRE at week 0. We assessed which MRE features were predictive for steroid-free remission at week 52, and which were associated with a secondary loss of response. 44 patients were in steroid-free remission at week 52, 17 - were secondary non-responders. The ROC curve showed that bowel thickening with contrast enhancement analyzed together at week 0 were associated with steroid-free remission at week 52 (p = 0.01; AUC 0.67). Bowel stenosis with or without prestenotic dilatation [OR 5.8 (95% CI 1.4 – 25) and 2.4 (95% CI 1.2 – 5) respectively; p = 0.01] and the presence of intra-abdominal fistulas [OR 1.4 (95% CI 1.1 – 2); p = 0.004] were related to secondary non-response. A high baseline inflammatory activity detected by MRE predicts one-year response in CD after IFX/ADA. In case of bowel stenosis, intra-abdominal fistulas, other therapeutic options should be considered.

The introduction of anti-tumor necrosis factor alpha (anti-TNF) antibodies to the treatment of inflammatory bowel diseases (IBD) is considered to be one of the most important advances in gastroenterologic therapeutics in recent years. It has significantly improved the therapeutic possibilities especially in Crohn’s disease (CD) and changed the understanding of new treatment goals (mucosal healing, deep remission, steroid-free remission) in IBD^1^. However, there are still many questions as to when and how to treat patients with anti-TNFs. An important limitation of anti-TNF therapy is the loss of response to treatment over time^2^. In order to optimize the therapy and to improve its efficacy, individualization of therapeutic schedules has been proposed. The measurement of drug trough levels and anti-drug neutralizing antibodies allows for the modification of the treatment algorithms, which can lead to better long-term therapeutic outcomes, higher mucosal healing rates and less surgery^3^. Another possibility of treatment optimization is the appropriate selection of patients for anti-TNF therapy. Several predictors of a good response to anti-TNF agents or treatment failure have been described^4^. However, results from different studies concerning this aspect of the optimization of anti-TNF therapy are conflicting.

The development of new cross-sectional imaging techniques such as magnetic resonance enterography (MRE) has, in recent years, significantly improved the possibilities of assessing the activity of the small bowel in CD^5^. One of the most important advantages of MRE is that it enables the visualization of the whole spectrum of inflammatory lesions in CD – endoluminal, mural and extramural. Thus, MRE is helpful in describing CD phenotype and behavior according to the CD Montreal classification, which defines the disease location in the gastrointestinal tract and differentiates between luminal, penetrating, and stricturing forms of the disease^6^. Moreover, the non-invasive nature of MRE and the lack of radiation exposure allow for the repeated performance of this investigation, thereby enabling the dynamic assessment of CD progression or regression in time. This is particularly important in the monitoring of patients undergoing anti-TNF therapy.

The usefulness of MRE in CD diagnostics has been proved in many studies^5^,^6^,^7^. Moreover, there is an increasing number of scoring systems quantifying CD activity in MRE^8^,^9^,^10^. It has been also shown, that MR imaging can be very helpful in monitoring anti-TNF therapy in CD patients^11^,^12^,^13^. However, little is known whether MRE assessment can be helpful in predicting the response to anti-TNF therapy. In this study, we performed a retrospective analysis of the possible role of MRE in predicting long-term and steroid-free remission in patients with CD treated with biological agents, who initially responded to induction doses of anti-TNF antibodies. We also analyzed which MRE parameters can predict secondary non-response in this group of patients.

## Results

Among 90 patients treated with anti-TNF antibodies, 61 (68%) were primary responders (40 treated with IFX and 21 treated with ADA) and they comprised the final study group. All further analyses concerning the usefulness of different radiological, and biochemical parameters in predicting one-year efficacy of anti-TNF therapy corresponded to this group of patients.

There was a slight predominance in the number of female CD patients, mean disease duration was 6 ± 4 years. Biochemical analyses showed elevated inflammatory markers, like C-reactive protein (CRP) or erythrocyte sedimentation rate (ESR). Median CDAI was 267 points (95%CI: 232 – 292), which corresponded to a moderate clinical activity of CD. Almost 40% of patients underwent surgery in the past because of CD. The majority of patients demonstrated an inflammatory phenotype of CD. Full characteristics of the whole study group is presented in Table 1.

44/61 (72%) patients reached the primary therapeutic end point, and 17/61 (28%) patients were classified as secondary non-responders. Secondary non-responders had significantly higher baseline CRP concentrations and higher platelet (PLT) counts when compared with patients who achieved steroid-free remission at week 52. Patients with longer disease duration and previous surgery were more probable to experience a secondary loss of response to anti-TNF therapy, however these differences were not statistically significant. In contrast to that, inflammatory phenotype of CD was significantly related to a higher probability of sustaining long-term remission at week 52 in comparison with penetrating and stricturing disease. Comparison between the two aforementioned study subgroups in terms of other biochemical, clinical, radiological and endoscopic features is presented in Table 2.

**Figure 1:**
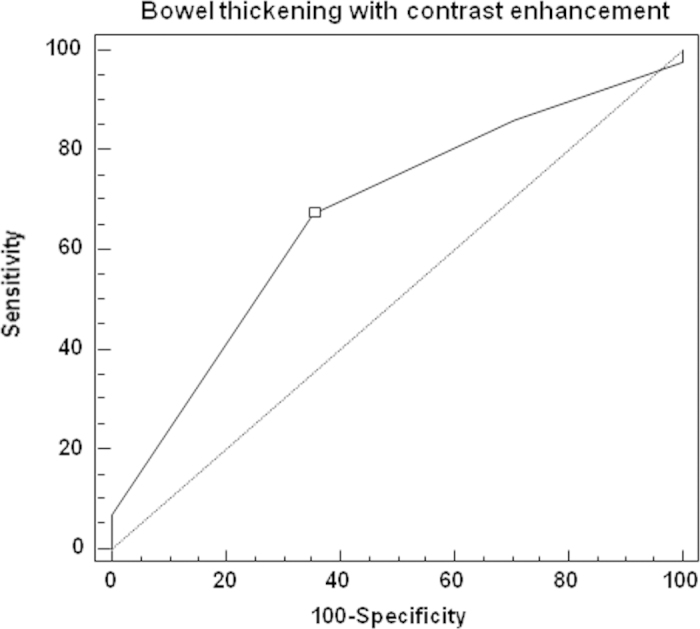
Receiver operator characteristic (ROC) curve showing that the score assessing bowel inflammatory thickening with contrast enhancement in the baseline magnetic resonance enterography (range 0 – 4 points) was predictive for long-term steroid-free remission at week 52 in Crohn’s disease patients treated with anti-TNF antibodies. An optimal cut-off point was ≥ 3, and it allowed to predict with 67% (confidence interval: 52 – 81%) sensitivity and 65% (confidence interval: 39 – 86%) specificity achievement of the primary therapeutic end point of the study (p = 0.01; area under the curve 0.67).

Calculation of ROC curves showed that none of the MRE features, analyzed separately, had a potential to predict one-year remission in the study group. However, when two features, which are routinely assessed together in daily practice – bowel wall thickening and contrast enhancement - were also statistically analyzed together (range 0 – 4 points), ROC curve showed that they had the potential to predict the achievement of primary therapeutic end point in the study group (p = 0.01; AUC 0.67). An optimal cut-off point was ≥3 points, with 67% (CI 52 – 81%) sensitivity and 65% (CI 39 – 86%) specificity (Fig. 1). Combination analyses of other investigated features did not show any statistical significance.

Binary logistic regression analyses revealed that bowel stenosis (p = 0.01) and the presence of intra-abdominal fistulas (p = 0.004) seen in MRE were significantly related to secondary non-response in the study group (Table 3). Other parameters were not related to a secondary treatment failure. In case of ulcerations the statistical analysis was impossible due to too few cases of ulcerations in the analyzed sample.

**Figure 2:**
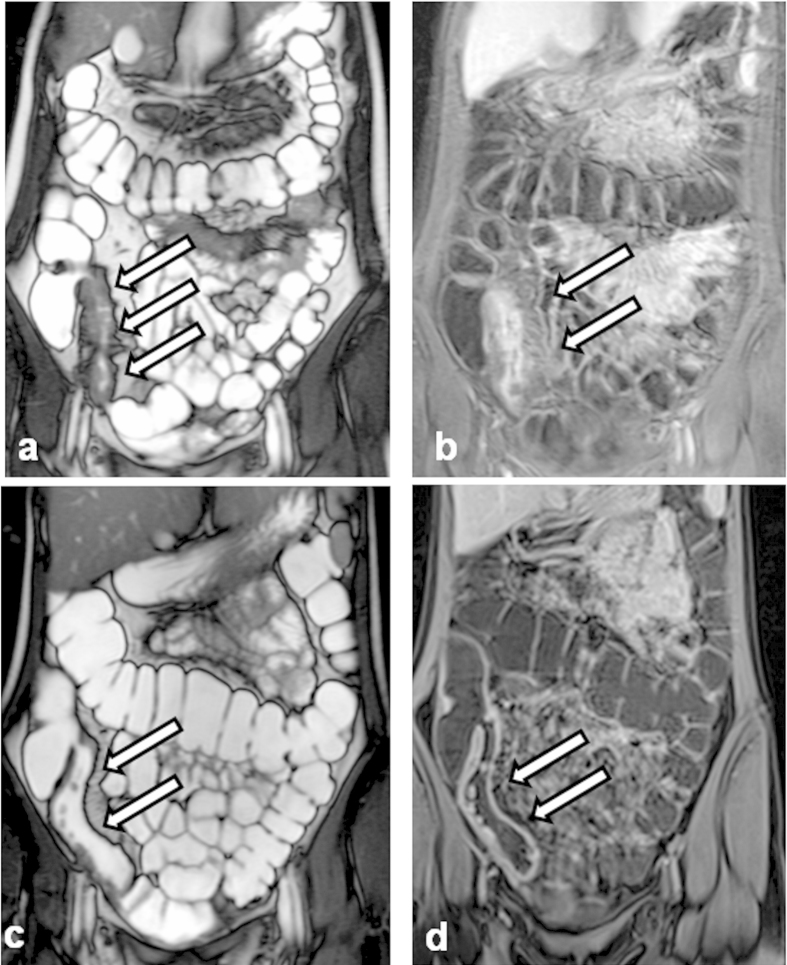
Magnetic resonance enterography images illustrating an example of a response to one-year anti-tumor necrosis factor (anti-TNF) therapy in a patient with Crohn’s disease: (**a**) Week 0 - before anti-TNF therapy. T2-weighted sequence demonstrating thickening of a bowel wall (white arrows) without stenosis of the bowel lumen. (**b**) Week 0 - before anti-TNF therapy. Dynamic contrast enhanced T1-volume interpolated gradient-echo sequence demonstrating thickening of a bowel wall with enhancing in the various layers of the wall - hyperintense signal corresponding to edema in acute inflammation (white arrows). (**c**) Week 52. T2-weighted sequence demonstrating decrease of thickening of the bowel wall (white arrows) after one-year anti-TNF therapy. (**d**) Week 52. Dynamic contrast enhanced T1-volume interpolated gradient-echo sequence demonstrating decrease of thickening of the bowel wall with moderate enhancement typical for moderate activity of inflammation (white arrows) - after one-year anti-TNF therapy.

**Figure 3:**
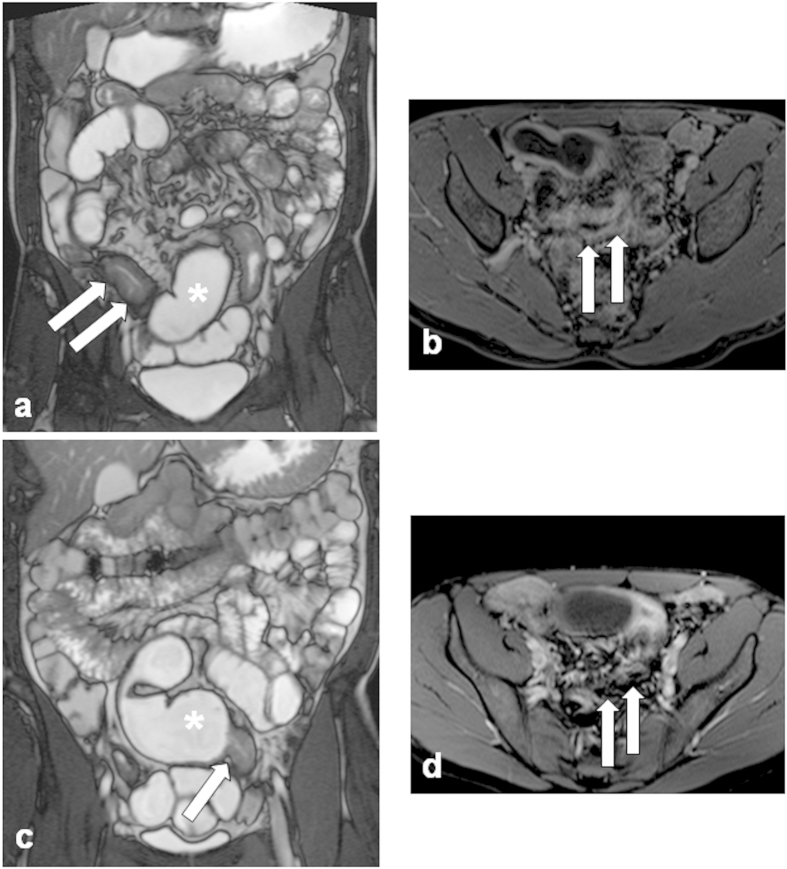
Magnetic resonance enterography images illustrating an example of a non-response to one-year anti-tumor necrosis factor (anti-TNF) therapy in a patient with Crohn’s disease: (**a**) Week 0 - before anti-TNF therapy. T2-weighted sequence demonstrating thickening of a bowel wall (white arrows) with stenosis of the bowel lumen with prestenotic dilatation (asterisk). (**b**) Week 0 - before anti-TNF therapy. Dynamic contrast enhanced T1-volume interpolated gradient-echo sequence demonstrating thickening of a bowel wall with enhancing in the various layers of the wall - hyperintense signal corresponding to edema in acute inflammation and dilatation of lumen (white arrows). (**c**) Week 52. T2-weighted sequence demonstrating increase of thickening of the bowel wall (white arrow) with stenosis of lumen and prestenotic dilatation (asterisk) after one-year anti-TNF therapy. (**d**) Week 52. Dynamic contrast enhanced T1-volume interpolated gradient-echo sequence demonstrating increase of thickening of the bowel wall with enhancement typical for activity of inflammation (white arrows) – after one-year anti-TNF therapy.

Figures 2 and 3 show MRE images illustrating examples of response and non-response to one-year anti-TNF therapy.

## Discussion

Clinical studies have shown that the initial response rate to anti-TNF therapy is approximately 60%. In this group, about 30% of patients maintained remission for up to one year^2^,^14^. Thus, appropriate qualification for biological therapy is essential in order to choose those patients who are the best candidates for anti-TNF treatment. Such patient selection is also important from a pharmacoeconomical point of view, and in order to minimize the risk of developing serious adverse reactions in subgroups of patients, who are already less likely to respond to anti-TNF therapy^15^.

Little is still known about which factors predict a good response or failure of anti-TNF therapy in CD. So far, several clinical, biochemical, and genetic parameters have been investigated in order to determine their association with response to anti-TNF agents. It has been hypothesized that the best candidate for anti-TNF therapy is a young, non-smoking patient with short CD duration, colonic disease location, non-stricturing CD behavior, having high CRP concentration and who had not been previously treated surgically^4^,^16^,^17^,^18^. Additionally it seems that the presence of perinuclear anti-neutrophil cytoplasmic antibodies (pANCA) and the absence of anti-Saccharomyces cerevisiae antibodies (ASCA) is associated with a failure of anti-TNF therapy^19^. The utility of several genetic factors in determining the expected efficacy of biologic agents is still questionable^4^,^20^. It should however be pointed out that the majority of the studies cited, referred to the usefulness of the various factors in predicting short-term response to anti-TNF therapy. Little is known about their usefulness in predicting long-term steroid-free remission of CD. We also do not know which factors could be associated with secondary loss of response to biological therapy

Among other investigations, MRE has in recent years become one of the most frequently used cross-sectional imaging methods in estimating CD behavior and activity^5^. Although it must be emphasized that, according to the consensus statement of the International Organization for the Study of Inflammatory Bowel Diseases (IOIBD), cross-sectional imaging methods (like MRE or ultrasound) have not been validated so far, current European Crohn’s and Colitis Organisation (ECCO) guidelines for imaging techniques in IBD indicate that they can be routinely applied for the assessment of the CD activity. It was also emphasized in the guidelines that MR techniques are especially useful as their diagnostic accuracy is similar to the accuracy of computed tomography, but with the major advantage of not imparting ionizing radiation^21^,^22^. Thus, it seems to be essential to investigate the usefulness of MRE in predicting anti-TNF treatment efficacy. To the best of our knowledge, our study is one of the first of its type to determine the predictive role of several imaging features seen in MRE in patients with CD qualified for anti-TNF therapy.

Several scores quantifying CD activity in MRE have been developed. The majority of them consist of similar MRE parameters, although none of those radiological features is pathognomonic for CD. Some of them can also be seen in other inflammatory small bowel disorders (mainly in gastrointestinal infectious diseases like yersiniosis), and non-inflammatory diseases, like for example small bowel neoplasms or ischemia^23^. That is why it is important to analyze all radiological features always in the clinical context. The most typical, major MRE features for CD are fat wrapping, an abnormal bowel wall thickness together with a mural hyperenhancement, enlargement and edema of mesenteric lymph nodes^5^. Another specific feature of CD – ulcerations, can be seen in the case of a full luminal distension, and it concerns only moderate to deep linear ulcers. Superficial, early ulcerations are not well demonstrated at MRE imaging.

In recent years, development of some new MRE sequences and methods for the assessment of CD activity has taken place. This increases the number of inflammatory parameters defined by MR imaging, among those discussed above. One of the most promising methods is the diffusion-weighted imaging (DWI). DWI reflects the changes in the water mobility caused by interactions with cell membranes, macromolecules, and alterations of the tissue environment^24^. It was shown that DWI enables detection of inflammatory activity in small bowel and colonic CD and is an accurate tool in assessing the disease activity. However, more studies are needed in this field.

One of the most well-known scores for “classical” MRE assessment in CD is the Magnetic Resonance Index of Activity (MaRIA) proposed by Rimola *et al.*^8^. MaRIA allows to assess the CD activity both in the small and the large bowel. This is due to the fact, that in the aforementioned study the colon was additionally filled with warm water by inserting a flexible rectal balloon catheter, which gave a sufficient large bowel distension^8^,^10^. This is in contrast with the majority of MRE protocols, in which MRE imaging is performed completely noninvasively, focusing on the assessment of the small bowel CD activity. Thus we decided to use a simple score allowing the assessment of CD activity in MRE performed according to a “standard” protocol^10^. In consequence, we also excluded from our study patients with isolated colonic CD location. The utility of the MRE score used in the current study was proved in an independent group of CD patients, and it correlated significantly with endoscopic activity, which was considered the gold standard for evaluation of CD activity. This score takes into account the most important and frequently assessed radiographic features of CD. Due to its simplicity, it can be easily applicable.

The statistical analysis revealed that an active inflammatory involvement of the intestine, reflected by bowel wall thickening and contrast enhancement in the baseline MRE were predictive of one-year steroid-free remission at week 52 in patients treated with anti-TNF agents. An optimal cut-off point was ≥3 points. This means that the best candidates for anti-TNF therapy are patients with bowel wall thickening ≥3 mm with a layered enhancement pattern after gadolinium administration or patients with bowel wall thickening >7 mm and any type of contrast enhancement. Additionally, the inflammatory (non-stenosing and non-penetrating) disease behavior was also significantly associated with long-term remission at week 52. These data confirm that high inflammatory burden of CD is associated with a good response to anti-TNF agents^25^,^26^. However, it is still a matter of debate how to define this inflammatory burden in the case of CD. Nevertheless, patients with all active forms of CD can benefit from anti-TNF therapy, but probably those with high luminal inflammatory activity without complications will have better therapeutic outcomes. Our study is one of the first studies to show that this observation can also be applied to MRE investigations. However, it should be pointed out that only a combined approach including at least two features of MRE activity – presence of lesions with increased contrast enhancement - might indicate positive long-term therapeutic effects.

Due to the increasing number of CD patients treated worldwide with anti-TNF antibodies, the problem of a secondary non-response becomes one of the most important limitations of this therapy^2^. Optimizing the treatment algorithm by increasing drug doses or by shortening the interval between doses, based on pharmacokinetic analyses, seems to be a valuable option in this clinical situation^3^,^27^,^28^. However, the wide application of drug trough levels monitoring and anti-drug antibodies testing in everyday practice is still not commonly accessible^3^. Thus, more attention should be paid to the proper selection of patients who have the highest probability of achieving long-term CD remission with biological treatment. Our analysis showed that patients with at least one stenosis with prestenotic bowel dilatation assessed in dynamic sequences had an almost 6-fold higher risk of developing a secondary non-response when compared with patients without stenosis. What is more, even the presence of stenosis without prestenotic dilatation was significantly associated with secondary anti-TNF treatment failure. It should be emphasized that none of the patients with stenoses detected in MRE had radiographic signs of their fibrotic etiology. Additionally none of the patients suffered from obstructive gastrointestinal symptoms before starting the anti-TNF therapy. Current recommendations allow the use of anti-TNF agents in patients with stenotic CD, but only if they are not of a fibrotic origin^29^,^30^. In fibrostenotic CD, endoscopic dilatation or surgical treatment should be considered^31^. Our study also shows the probability that patients with non-fibrotic stenoses should not be treated with anti-TNF agents, as they are at a high risk of developing a secondary non-response. Alternatively a more individualized and aggressive treatment algorithm should be introduced in this subgroup of patients, in order to avoid surgery.

The presence of intra-abdominal fistulas detected in MRE increased also the risk of developing secondary non-response to anti-TNF therapy. Current guidelines do not recommend pharmacological treatment in CD patients with intra-abdominal fistulas, as they should be treated surgically^31^. Unfortunately, this recommendation cannot always be easily applied in everyday clinical practice. Some patients do not accept surgical treatment, especially when they have undergone one or more bowel resections in the past. In our cohort of patients with intra-abdominal fistulas who initially responded to anti-TNF therapy and then developed secondary non-response (n = 6), all of them either did not initially accept the surgical treatment option or were disqualified from surgery because of other co-morbidities. After secondary failure of anti-TNF therapy, the majority of them (4/6) were treated surgically, and two received total parenteral nutrition (data not shown). Thus, since the efficacy of anti-TNF therapy in patients with intra-abdominal fistulas is poor, it seems that more attention should be paid in convincing patients to undergo surgical or alternative treatment.

Another observation from our study is that fat wrapping, which is believed to be one of the most characteristic features of an active CD, was not predictive for long-term therapeutic outcomes^5^. However, the grading score applied for this parameter could be less discriminative in order to differentiate active from inactive inflammatory lesions in MRE. That is why, as this parameter is always assessed together with vascular proliferation in the mesenteric fatty tissue, in order to make this aspect of CD activity estimation in MRE more discriminative, we summed up (scoring range 0–3 points) these two strictly interrelated parameters (fat wrapping and vascular proliferation in the mesenteric fatty tissue). In the first step, we performed an additional statistical analysis in which we divided our study group into three subgroups depending on their BMI results: <18.5 kg/m^2^ (n = 9), 18.5 – 25 kg/m^2^ (n = 45), and >25 kg/m^2^ (n = 7). Comparative analysis revealed no differences in the intensity of fat wrapping and vascular proliferation in those subgroups. Then, after checking that nutritional status does not interfere with the assessment of the discussed MRE parameters, we conducted further statistical analysis in order to estimate whether fat wrapping and vascular proliferation can be predictive for long-term treatment outcomes. The analysis, however, did not show their usefulness to predict the long-term response in our study group (OR for 2 points = 1; 95%CI 0.6 – 1.7, OR for 3 points = 1.3; 95% CI 0.04 – 37.1; p = 0.4). That is why we hypothesize that although this parameter is important for the assessment of CD activity, it seems probably not to have prognostic value.

Analysis of biochemical data also showed, that patients who were secondary non-responders had a significantly higher baseline CRP concentration. This seems to be in contrast with the results of some other studies. Louis *et al.* were first to show that a higher CRP level before treatment was associated with a positive clinical response to IFX^25^. Several other studies revealed that high baseline CRP concentration and its normalization after induction doses of anti-TNF agents were predictive of maintained clinical efficacy of biological therapy^15^,^26^,^32^. On the other hand it has been shown that high CRP levels at the moment of diagnosis are associated with a worse CD course and that they predict the need for surgical treatment^33^. The most recent study by Magro *et al.* also showed that a high CRP concentration predicts rather a non-response to therapy with IFX in CD^34^. Thus, it should be emphasized that data in terms of the predictive role of CRP in anti-TNF therapy are conflicting. One can speculate, that the reason for these differences is probably due to differences in the etiology of CRP elevation. High inflammatory burden reflected by high CRP level is connected with a better outcome of anti-TNF therapy. The presence of CD complications (fistulas, stenoses) which can also lead to CRP elevation is related to a worse efficacy of biological agents. This hypothesis can also explain why patients with a higher baseline PLT count more frequently experienced a secondary non-response to anti-TNF therapy in our study group, as PLT are believed to be one of the most sensitive markers of CD severity^35^. However, it should be mentioned that although biochemical markers can be helpful, current guidelines emphasize the need of more complex assessment of CD activity by using endoscopy and cross-sectional imaging methods, which allow to estimate the whole spectrum of mucosal and transmural damage^36^.

Our study has several limitations. The study group was strictly defined and the analyses concerned only primary responders to anti-TNF therapy, thus our data might be biased. Moreover, the number of patients is relatively low, which is due to very strict inclusion and exclusion criteria. This potentially could lead to some statistical misinterpretation of obtained data. Thus, although our study group is still one of the largest in the discussed subject matter, there is an urgent need to confirm the results in larger, prospective trials.

Besides, it is still not fully clear which MRE features should be used in the first line in the assessment of CD activity, and how some of them should be interpreted. Some studies in that matter bring conflicting results. For example it is still being discussed how to define and stratify bowel wall thickening. Cutoff points for differentiation of different CD stages and its activity are still lacking or data are conflicting^5^. For example, there are some data using 4 mm as a cut off point for an abnormal bowel wall thickness^37^. It was also shown, that MRE was accurate for grading mural small bowel CD mainly in case of frank disease, and there could be some disease overstaging in the cases of patients in remission or mild CD activity^5^,^38^. Another unanswered question is whether bowel wall thickness requires an adjustment for different body weight or previous abdominal surgery. As there are no data in this field, we tried to analyze if these factors had an impact on CD mural lesions. We did not find any correlation between patients‘ BMI and bowel wall thickness. Moreover, we divided patients according to their BMI into three groups: <18.5 kg/m^2^ (n = 9), 18.5 – 25 kg/m^2^ (n = 45), and >25 kg/m^2^ (n = 7). Additional statistical analysis showed that bowel wall thickness was similar when compared patients with a different BMI. We also divided our study group into patients who underwent surgery in the past, and those without surgery. The median bowel wall thickness was 2 (95%CI 1.5–2.1) and 2 (95%CI 1.7–2) respectively, so the difference was not significant (p = 0.5). That is why we hypothesize that BMI or previous surgery do not influence this MRE parameter. Moreover, in the study performed by Gallego-Ojea *et al.* in which the usefulness of MRE in the detection of disease recurrence after surgery was shown, the authors also considered bowel wall thickness ≥3 mm as abnormal^39^. That is why, according to the current guidelines and teaching tutorials for radiologists, we decided to consider bowel wall thickness ≥3 mm as abnormal although some controversies in this field still exist^40^.

Additionally, heterogeneous enhancement of the bowel wall after contrast administration, believed to be a sign of active mural inflammation, was also suggested to be associated with fibrostenotic disease^5^,^41^. Moreover, in our study contrast enhancement was rated on a qualitative score. It is also unknown, whether characteristics of an individual patient can interefere with the reading of hyperenhancement in MRE. Although there are no data in this field, we divided our patients once again, depending on the BMI results, into three group, as presented above, and performed an additional statistical analysis. We did not find any association bewteen BMI and the character of contrast enhancement (data not shown). Thus, we hypothesize that this parameter is not related to patients‘ nutritional status.

To summarize this part of the discussion, we would like to emphasize that all selected MRE features assessed by the radiologist in our study are believed to be the most sensitive in detecting inflammatory activity of CD^5^,^6^,^7^,^8^,^9^,^10^. Despite some of the controversies presented above, MRE is still a method of choice in diagnosing and monitoring small bowel CD, and the majority of MRE assessment protocols used worldwide consist of very similar features as those assessed in our study.

The most important limitation of our study is that our MRE images were assessed by a single radiologist, thus, there was no interobserver agreement analysis performed. Moreover, a qualitative assessment of MRE images can be subjective, especially in the case of wall thickness and hyperenhancement quantitation. However, other studies have shown that the agreement between experienced radiologists in MR assessment is high, especially in large university radiological centers^42^. We would also like to emphasize that our radiologist is highly experienced in this type of MRE technique and was blinded to the anti-TNF efficacy results.

In summary it should be pointed out that MRE is an adequate tool not only in assessing CD extent and its activity. This cross-sectional imaging technique can also provide valuable prognostic data regarding the expected efficacy of anti-TNF treatment in a CD patient. Thus, it could be helpful in optimizing biological therapy. One of the main limitations of more frequent application of MRE is its relatively high cost. According to British data, the mean cost of MR enteroclysis is £268 and is slightly higher than MRE (£241). CT enterography seems to be the cheapest method, as its cost is £133^43^. Although costs can differ between countries and even between different departments, greater financial burden of MR imaging is a rule. Thus, some authors suggest that MRE should be performed only in the youngest patients and in other cases CT enterography is preferred. It seems, however, that a proper qualification for anti-TNF therapy can lead to the optimization of this expensive treatment and can lower its costs. Thus, as MRE is believed to be a useful method in the assessment of CD activity, the application of this method in qualification process for anti-TNF therapy could, hypothetically, be economically beneficial^22^,^23^. The reason for this is that selecting the best candidates for anti-TNF treatment is still challenging, as approximately 30% of all CD patients will lose the initially obtained response to this therapy. Our study showed for one of the first times that MRE is probably useful in this selection process. This could limit toxicity, save costs and allow for the more accurate use of anti-TNF agents. However, in order to undoubtedly confirm these data, and to allow to directly translate them into clinical practice, there is a need for larger prospective trials in which an additional interobserver agreement analysis would be performed, before more widespread implementation of MRE in patients with CD will take place.

## Methods

### Study population

A retrospective cohort study was performed among patients with CD hospitalized between 2009 and 2012 at the Department of Gastroenterology, Human Nutrition and Internal Diseases at Poznan University of Medical Sciences. The inclusion criteria were as follows:

age ≥18 years

exacerbation of CD ineffectively treated with steroids and/or immunomodulators and meeting the eligibility criteria for anti-TNF therapy

realisation of MRE in accordance with the same protocol (presented below) 1–14 days before starting anti-TNF therapy

response to the induction regimen of anti-TNF therapy with infliximab (IFX) or adalimumab (ADA) according to the criteria defined below, allowing for the continuation of maintenance therapy.

The exclusion criteria were:

primary non-response to anti-TNF therapy

need for any change in treatment regimen during induction doses of anti-TNF therapy

isolated colonic location of CD, in which the diagnostic usefulness of MRE is lower than in ileal or ileocolonic CD

treatment with IFX or ADA within 52 weeks before enrollment into the study

presence of any general contraindications to MR procedure, such as any metallic medical devices or foreign bodies (for example cardiac pacemaker, implanted cardioverter-defibrillator, joint replacement etc.) or renal insufficiency.

The clinical activity was routinely assessed by calculating the Crohn’s Disease Activity Index (CDAI)^44^. Each patient also underwent ileocolonoscopy performed by an experienced endoscopist (KL, IKK, LLS) with the estimation of the Simple Endoscopic Score for Crohn’s Disease (SES-CD)^45^. Biochemical markers of CD activity were measured routinely.

### MRE protocol

MRE examinations were performed with a 1,5 Tesla clinical unit (Magnetom Avanto, Siemens Medical Solutions, Erlangen, Germany) with six-channel abdominal phased-array coils with a spine matrix coil. Each patient underwent MRE according to the same standard protocol previously described^10^. Namely, patients fasted for 6 hours before MRE. Patients were then given 1500 ml of oral polyethylene glycol (PEG) electrolyte solution (a single sachet of PEG diluted in 1500 ml of water – Fortrans, Ipsen Pharma) to drink 30–40 minutes before the investigation. Additionally, 40 mg of buscolysin (Hyoscini butylbromidum, Sopharma Plc, Bulgaria) was given intravenously prior to gadolinium administration in order to reduce bowel motility. To reduce respiratory movements and provide higher image quality, all imaging was performed under breath-hold conditions. The standard study protocol consisted of the sequences listed in Table 4.

### Image analysis

The most important and best studied MRE features of CD activity were assessed and quantified according to the scale proposed in Table 5^6^,^7^,^8^,^9^,^10^. The usefulness of our score was proved in an independent cohort of CD patients, where it was compared with endoscopy, which is thought to be the gold standard in the assessment of CD activity^10^. The evaluation of MRE was performed by a single radiologist (KK), who was blinded to the efficacy results of anti-TNF therapy, and who has more than 10 years of experience in this imaging method.

### Anti-TNF treatment regimens and definition of therapeutic end points

According to the anti-TNF induction regimen, patients were then given either 3 doses of 5 mg/kg body weight of IFX intravenously at week 0 – 2 – 6, or 160 mg of ADA subcutaneously at week 0, 80 mg at week 2 and 40 mg every other week until week 12.

Follow-up investigations, including ileocolonoscopy, were performed at week 10 in case of IFX and at week 13 in case of ADA. Primary response to anti-TNF therapy was defined as a ≥100 – point decrease in the CDAI score (CDAI-100 response) with concomitant endoscopic improvement (any decrease in the SES-CD score). All primary responders were then placed on the maintenance regimen: 5 mg/kg body weight IFX every 8 weeks or 40 mg ADA every other week until week 52.

The therapeutic primary end point was clinical and steroid-free remission (CDAI < 150 points) at week 52. All patients who did not reach the therapeutic primary end point (CDAI ≥ 150 points and/or need for reintroducing steroids), and who needed optimization of anti-TNF therapy (increasing the doses or decreasing the intervals between doses) were considered as secondary non-responders. The main aim of the study was to assess which of the MRE parameters assessed during baseline imaging study have the potential to predict one-year steroid-free remission or secondary non-response in patients treated with anti-TNF antibodies.

### Statistical analysis

Descriptive statistics was made by calculating means with standard deviations or medians with 95% confidence intervals. To compare clinical, endoscopic and biochemical data, the Student t test (parametric) was used when conditions of normality and equal variance were met. The Student t test was used with Welch correction when unequal variances were detected. When the normality test failed, a 2-tailed and exact Mann–Whitney rank sum test (nonparametric) was used. The differences in the disease behavior and disease locations in the study subgroups were assessed by using the Chi square test.

Each MRE feature was graded according to the scoring system presented in Table 5 and assessed statistically separately, and – in the next step - in combination with other MRE features. In the second case the results were summarized. Receiver operating characteristic (ROC) curves were calculated to determine the potential of analyzed parameters to differentiate patients who had reached the therapeutic primary end point. An optimal cut-off point was calculated according to the highest accuracy (minimal false negative and false positive results). The area under the ROC curve (AUC) was used to check the prognostic value of particular parameters. For parameters which showed prognostic value, their sensitivity and specificity were additionally calculated.

In order to determine parameters which could be predictive of secondary loss of response to anti-TNF agents, a binary logistic regression analysis with subsequent cross validation was performed to identify the best discriminating MRE parameters by calculating the percentage overall correct classifications. For all studied parameters, odds ratios (OR) and 95% confidence intervals (CI) were presented.

Statistical analyses were performed with MedCalc version 10.3.2 (MedCalc Software, Mariakerke, Belgium) and Statistica 10 (StatSoft Inc., Poland). All tests were considered significant at a p value of less than 0.05.

The study was approved by the Bioethics Committee at the Poznan University of Medical Sciences (No 774/13). An informed consent was obtained from all patients. The protocol of this study was performed strictly in accordance with the approved guidelines.

## Author Contributions

P.E. designed the study, carried out the study and data analyses and drafted the manuscript. M.M. performed the statistical analyses and helped to draft the manuscript. L.L.S., K.K., I.K.K., K.S.E. helped to design and to revise the manuscript. K.K., A.S. and K.L. helped to revise and drafted the manuscript. All authors read and approved the final manuscript.

## Additional Information

**How to cite this article**: Eder, P. *et al.* Magnetic resonance enterographic predictors of one-year outcome in ileal and ileocolonic Crohn’s disease treated with anti-tumor necrosis factor antibodies. *Sci. Rep.* **5**, 10233 doi: 10.1038/srep10223 (2015).

## Figures and Tables

**Table 1:** Baseline characteristics of the 61 Crohn’s disease patients, who responded to the induction anti-tumor necrosis factor therapy (primary responders), which comprised the final study group.

**Feature**	**n=61**
Age [mean ± SD]	32 ± 10
Male/female	29/32
Disease duration (years) [mean ± SD]	6 ± 4
C-reactive protein (mg/l) [mean ± SD]	22.1 ± 26.1
Erythrocyte sedimentation rate (mm/h) [mean ± SD]	32 ± 20
Red blood cell count (10^6^/mm^3^) [mean ± SD]	4.4 ± 0.7
Hemoglobin (g/dl) [mean ± SD]	12.3 ± 2.2
Hematocrit (%) [mean ± SD]	37 ± 5
White blood cell count (10^3^/mm^3^) [mean ± SD]	7.5 ± 3.7
Platelets (10^3^/mm^3^) [mean ± SD]	358 ± 109
Body mass index (kg/m^2^) [mean ± SD]	20.9 ± 3.3
Crohn’s Disease Activity Index [median (95%CI)]	267 (232 – 292)
Simple Endoscopic Score for CD (SES-CD) [median (95%CI)]	12 (11 – 18)
Ileal SES-CD [median (95%CI)]	5 (4 – 7)
Colonic SES-CD [median (95%CI)]	8 (7 – 13)
Previous surgery – n (%)	24/61 (39%)
Disease location – n (%)	
L1 (ileal)	26/61 (43%)
L3 (ileocolonic)	35/61 (57%)
Disease behavior – n (%)	
B1 (inflammatory)	32/61 (53%)
B2 (stricturing)	10/61 (16%)
B3 (penetrating)	19/61 (31%)
Medications	
Steroids	26/61 (42%)
Azathioprine	53/61 (87%)
Aminosalicylates	60/61 (98%)
Probiotics	46/61 (75%)
Antibiotics	15/61 (24%)
Previous anti-TNF therapy	8/61 (13%)

Data are presented as means with standard deviations (SD) or medians with 95% confidence intervals (CI).

**Table 2:** Comparison of baseline data (week 0) of Crohn’s disease patients who achieved one-year steroid-free remission at week 52 (responders) with secondary non-responders group.

**Feature**	**Responders**	**Non-responders**	**p**
Number of patients (%)	n=44/61 (72%)	n=17/61 (28%)	
Age (years) [mean ± SD]	30 ± 8	37 ± 13	ns
Male/female –n (%)	22/22	7/10	
Disease duration (years) [mean ± SD]	5 ± 3	6 ± 5	ns
C-reactive protein (mg/l) [mean ± SD]	16.4 ± 15.7	35.3 ± 38.7	0.01
Erythrocyte sedimentation rate (mm/h) [mean ± SD]	30 ± 18	37 ± 23	ns
Red blood cell count (10^6^/mm^3^) [mean ± SD]	4.4 ± 0.8	4.4 ± 0.7	ns
Hemoglobin (g/dl) [mean ± SD]	12.5 ± 2.1	11.6 ± 2.5	ns
Hematocrit (%) [mean ± SD]	38 ± 5	36 ± 6	ns
White blood cell count (10^3^/mm^3^) [mean ± SD]	7.3 ± 3.5	7.9 ± 4.2	ns
Platelets (10^3^/mm^3^) [mean ± SD]	328 ± 98	429 ± 103	0.009
Body mass index (kg/m^2^) [mean ± SD]	21.2 ± 3	20.9 ± 3.3	ns
Crohn’s Disease Activity Index [median (95%CI)]	264 (220 – 294)	304 (216 – 331)	ns
Simple Endoscopic Score for CD (SES-CD) [median (95%CI)]	12 (11 – 18)	12 (4 – 25)	ns
Ileal SES-CD [median (95%CI)]	6 (4 – 7)	6 (3 – 6)	ns
Colonic SES-CD [median (95%CI)]	9 (6 – 12)	12 (11 – 18)	ns
Disease location – n (%)			
L1 (ileal)	18/44 (41%)	9/17 (53%)	ns
L3 (ileocolonic)	26/44 (59%)	8/17 (47%)	ns
Disease behavior – n (%)			
B1 (inflammatory)	27/44 (61%)	5/17 (29%)	0.04
B2 (stricturing)	6/44 (14%)	4/17 (24%)	ns
B3 (penetrating)	11/44 (25%)	8/17 (47%)	ns
Previous surgery – n (%)	15/44 (34%)	9/17 (53%)	ns
Medications			
Steroids	21/44 (48%)	5/17 (29%)	ns
Azathioprine	39/44 (87%)	14/17 (82%)	ns
Aminosalicylates	43/44 (98%)	17/17 (100%)	ns
Probiotics	33/44 (75%)	13/17 (76%)	ns
Antibiotics	8/44 (18%)	7/17 (41%)	ns
Previous anti-TNF therapy	5/44 (11%)	3/17 (18%)	ns

Data are presented as means with standard deviations (SD) or medians with 95% confidence intervals (CI).

**Table 3:** Binary logistic regression analysis showing which features of Crohn’s disease activity assessed in magnetic resonance enterography at week 0 were associated with a secondary non-response in patients treated with anti-TNF antibodies. Data are presented as odds ratios (OR) with 95% confidence intervals (CI).

**Feature**	**Statistical significance in predicting secondary non-response to anti-TNF therapy**	**The percentage overall correct classifications**
Bowel wall thickening	1 point: OR 0.6; 95% CI (0.2 – 1.7); ns	—
	2 points: OR 0.1; 95% CI (0.001 – 10); ns	
Contrast enhancement	1 point: OR 1.1; 95% CI (0.4 – 25); ns	—
	2 points: OR 1.1; 95% CI (0.04 – 33.3); ns	
Fat wrapping	OR 0.9; 95% CI (0.3 – 2.5); ns	—
Proliferation of mesenteric vasculature	1 point: OR 1.2; 95% CI (0.6 – 2.5); ns	—
	2 points: OR 1.6; 95% CI (0.1 – 25); ns	
Lymphadenopathy	1 point: OR 1.1; 95% CI (0.5 – 2); ns	—
	2 points: OR 1.2; 95% CI (0.07 – 20); ns	
Bowel wall ulcerations	Analysis impossible due to too few cases of ulcerations (4 cases, all in the long-term responders group) in analyzed sample	—
Stenosis	1 point: OR 2.4; 95% CI (1.2 - 5); p = 0.01	75.40%
	2 points: OR 5.8; 95% CI (1.4 – 25); p = 0.01	
Intra-abdominal fistulas	OR 1.4; 95% CI (1.1 – 2); p = 0.004	75,5%
Disease extent	1 point: OR 0.6; 95% CI (0.3 – 1.25); ns	—
	2 points: OR 0.4; 95% CI (0.08 – 1.6); ns	

**Table 4:** Sequences used in the magnetic resonance enterography protocol.

	**Repetition time/echo time (ms)**	**Flip angle**	**Slice thickness (mm)**	**In plane resolution (mm)**	**Distance factor (Axial) (mm)**	**Distance factor (Coronal)(mm)**
True Fast Imaging with Steady-state free Precession (FISP)	4/1,72	60°	4,0	2,0 × 1,5	0	0,4
Single shot turbo spin echo sequence with fat suppression (HASTE)	1100/116	150°	6,0	1,5 × 1,6	—	1,8
Retro Steady State Free Precession	42,6/1,2	73°	6,0	1,5 × 2	—	6,0
Fat-suppressed 3D T1-weighted Volumetric Interpolated Breath-Hold Examination (VIBE) before and three times - 30, 90 seconds and 5 minutes after intravenous injection of gadolinium contrast (Gadovist 1.0, Bayer Pharma AG, Germany, dose of 0.1 mmol/kg body weight followed by 20 ml of saline)	6,10/2,74	10°	1,75	2 × 2	0,6	0,6

**Table 5:** Quantifying score of magnetic resonance enterographic features of Crohn’s disease activity 10.

**MRE feature**	**Grading scale**		
Bowel wall thickening	<3 mm: 0 pts	3 – 7 mm: 1 pt	>7 mm: 2 pts
Contrast enhancement^1^	None: 0 pts	Homogenous pattern: 1 pt	Layered pattern: 2 pts
Fat wrapping	None: 0 pts	Present: 1 pt	
Proliferation of mesenteric vasculature	None: 0 pts	<5 vessels/3 cm^2^ of mesenteric fat: 1 pt	≥ 5 vessels/3 cm^2^ of mesenteric fat: 2 pts
Mesenteric lymphadenopathy	None: 0 pts	<10 enlarged (diameter > 5 mm) lymph nodes: 1 pt	≥ 10 enlarged (diameter > 5 mm) lymph nodes: 2 pts
Bowel wall ulcerations	None: 0 pts	At least one ulceration present, not exceeding ½ of bowel thickness: 1 pt	At least one ulceration present, exceeding ½ of bowel thickness: 2 pts
Stenotic complications^2^	None: 0 pts	Stenosis without prestenotic dilatation: 1 pt	At least one stenosis with prestenotic dilatation: 2 pts
Intra-abdominal fistulas	None: 0 pts	At least one intra-abdominal fistula tract visible: 2 pts	
Extent of the disease in jejunum or ileum	<1500 mm: 1 pt		>1500 mm: 2 pts

^1^assessed in comparison with the adjacent bowel loops.

^2^decrease in bowel caliber in comparison with the adjacent bowel loops, with dilatation of the proximal segment and/or a collapse of the distal segment.
